# A Systematic Review of Interventions to Improve HPV Vaccination Coverage

**DOI:** 10.3390/vaccines9070687

**Published:** 2021-06-23

**Authors:** Edison J. Mavundza, Chinwe J. Iwu-Jaja, Alison B. Wiyeh, Blessings Gausi, Leila H. Abdullahi, Gregory Halle-Ekane, Charles S. Wiysonge

**Affiliations:** 1 Cochrane South Africa, South African Medical Research Council, Francie van Zijl Drive, Parow Valley, Cape Town 7501, South Africa; Charles.Wiysonge@mrc.ac.za; 2 Department of Nursing and Midwifery, Stellenbosch University, Francie van Zijl Drive, Tygerberg, Cape Town 7505, South Africa; chinwelolo@gmail.com; 3 Department of Epidemiology, University of Washington, Seattle, WA 98195, USA; wberiliy@yahoo.co.uk; 4 Division of Epidemiology and Biostatistics, School of Public Health and Family Medicine, University of Cape Town, Anzio Road, Observatory, Cape Town 7925, South Africa; sibusiso.gausi@alumni.uct.ac.za; 5 African Institute for Development Policy, Nairobi P.O. Box 14688-00800, Kenya; leylaz@live.co.za; 6 Faculty of Health Sciences, University of Buea, Buea P.O. Box 63, Cameroon; halle-ekane.edie@ubuea.cm; 7 Division of Epidemiology and Biostatistics, Department of Global Health, Stellenbosch University, Francie van Zijl Drive, Tygerberg, Cape Town 7505, South Africa

**Keywords:** human papillomavirus, vaccination coverage, recipient-oriented interventions, provider-oriented interventions, systematic review

## Abstract

Human papillomavirus (HPV) infection is the most common sexually transmitted infection worldwide. Although most HPV infections are transient and asymptomatic, persistent infection with high-risk HPV types may results in diseases. Although there are currently three effective and safe prophylactic HPV vaccines that are used across the world, HPV vaccination coverage remains low. This review evaluates the effects of the interventions to improve HPV vaccination coverage. We searched the Cochrane Central Register of Controlled Trials, PubMed, Web of Science, Scopus, and the World Health Organization International Clinical Trials Registry Platform and checked the reference lists of relevant articles for eligible studies. Thirty-five studies met inclusion criteria. Our review found that various evaluated interventions have improved HPV vaccination coverage, including narrative education, outreach plus reminders, reminders, financial incentives plus reminders, brief motivational behavioral interventions, provider prompts, training, training plus assessment and feedback, consultation, funding, and multicomponent interventions. However, the evaluation of these intervention was conducted in high-income countries, mainly the United States of America. There is, therefore, a need for studies to evaluate the effect of these interventions in low-and middle-income countries, where there is a high burden of HPV and limited HPV vaccination programs.

## 1. Introduction

Human papillomavirus (HPV) infection is the most common sexually transmitted infection worldwide [1]. It is estimated that 75% of sexually active men and women will acquire HPV infection in their lifetime. HPV infections are most prevalent in young adults, as sexual risk behaviors are greatest in this age group. Sexually active young women, in particular, carry the highest risk of infection, with studies documenting rates as high as 68–71% [2]. To date, more than 200 HPV types have been identified and classified into two groups: high-risk and low-risk types [3]. Although most HPV infections are transient and asymptomatic, persistent infection with high-risk HPV types may result in cancers, including cervical, anal, vulvar, vaginal, penile, and oropharyngeal cancers [4,5,6], and genital warts [6]. High-risk HPV types, including HPV-16, -18, -31, -33, -35, -39, -45, -51, -52, -56, -58, and -59 are associated with cancers in humans, whereas low-risk HPV types, including HPV-6, -11, -40, -42, -43, -44, -54, -61, and -72 cause benign diseases such as genital warts [7]. Among these HPV types, the majority of HPV-related clinical diseases are associated with HPV-16, -18, -6, and -11. HPV types 16 and 18 cause approximately 70% of cervical cancer, and HPV-6 and HPV-11 are responsible for approximately 90% of genital warts. Most HPV-associated morbidity and mortality is due to cervical cancer, the fourth most common cancer in women worldwide, with an estimated 604,127 cases and 341,831 deaths in 2020 [8]. HPV vaccination is an important tool to prevent and control HPV infection and its complications [5]. There are currently three prophylactic HPV vaccines that are used across the world: Cervarix, a bivalent HPV vaccine that targets HPV-16 and -18; Gardasil, a quadrivalent HPV vaccine that targets HPV-6, -11, -16, and -18; and Gardasil 9, a nonavalent HPV vaccine that targets HPV-6, -11, -16, -18, -31, -33, -45, -52, and -58 [9]. All three vaccines have proven to be highly efficacious against persistent infection of their vaccine genotypes. However, HPV vaccines are most effective when administered before debut and exposure to HPV [10]. HPV vaccination is currently recommended for adolescent males and females aged 9–14 years in a two-dose series and as a three-dose series for young men and women aged 15–26 years [11]. 

Despite its effectiveness, safety, and recommendations, HPV vaccination coverage remains low. Numerous barriers to HPV vaccination have been identified, including lack of health care provider recommendations, concerns about safety, concerns about side effects, and a general lack of awareness and knowledge about HPV vaccination [12]. There is, therefore, an urgent need for effective interventions to improve HPV vaccination coverage and reduce the burden of HPV-associated infections and cancers. Several reviews have assessed interventions to improve HPV vaccination coverage. However, the reviews assessed the effectiveness of interventions among adolescents [13], young adults [14], adolescents and young adults [15], the effectiveness of practice- and community-based interventions [6], and communication technology interventions [16]. A comprehensive systematic review on interventions to increase HPV vaccination coverage was published in 2016 [17]. However, the review included only studies conducted in the United States of America. Therefore, this review’s findings may not be applicable to low- and middle-income countries, where the burden of HPV is high, and vaccination coverage is very low. In addition, the review included only studies up to 2015, while there have been numerous potentially eligible studies published since then. To the best of our knowledge, there is no comprehensive systematic review that has assessed interventions to improve HPV vaccination coverage across all country income categories. These limitations justify the need for a comprehensive systematic review on the interventions to improve HPV vaccination coverage. 

## 2. Materials and Methods

The protocol for this review was registered in the International Prospective Register of Systematic Reviews (PROSPERO) (CRD42019138971) [18], and the review was prepared according to the Preferred Reporting Items for Systematic Reviews and Meta-Analyses (PRISMA) guideline [19].

### 2.1. Criteria for Considering Studies for This Review

We included randomized trials, non-randomized trials, interrupted time-series studies, and controlled before–after studies that met the quality criteria used by the Cochrane Effective Practice and Organization of Care (EPOC) [20]. We only included cluster-randomized controlled trials with at least two intervention and two control clusters. Interrupted time-series studies were only included if their outcomes were measured during at least three points before and after the intervention. We also included controlled before–after studies only if they had at least two intervention groups and at least two comparable control groups. We included studies conducted among all individuals eligible for HPV vaccines and their parents/legal guardians or healthcare providers. Included studies evaluated recipient-oriented, provider-oriented, legislative, health system, and multi-component interventions. Eligible studies compared the interventions to standard HPV vaccination practices, alternative interventions, or similar interventions implemented with different degrees of intensity. Our primary outcome of interest was HPV vaccination coverage, while our secondary outcomes were adverse effects and the cost of the intervention.

### 2.2. Search Methods for Identification of Studies

We developed a comprehensive search strategy with the help of an information specialist. We searched the following databases: the Cochrane Central Register of Controlled Trials (CENTRAL), PubMed, Web of Science, and Scopus. We searched databases from inception until the day of the search. We searched for published articles with no language restriction. We provided the search strategies for databases searched (Appendix A, Table A1). We also searched the WHO International Clinical Trials Registry Platform for ongoing trials and the reference lists of included studies and related reviews for other relevant studies. In addition, we searched the abstracts of the latest conferences of relevant scientific societies related to vaccination and HPV virology for new or pending information not yet published in peer-reviewed journals.

### 2.3. Selection of Studies 

Two review authors (Edison Mavundza [EM] and Chinwe Iwu-Jaja [CI]) independently screened the titles and abstracts to identify potentially eligible studies. Disagreements between the two authors were resolved by discussion and consensus. We obtained the full texts of all potentially eligible studies. Two authors independently screened the full texts and identified included studies, resolving discrepancies through discussion and consensus. Excluded studies are described in the table of excluded studies alongside their reasons for exclusion.

### 2.4. Data Extraction and Management

Two review authors (EM and CI) independently extracted data from each included study using a structured and standardized data extraction form. Extracted data included study setting, type of study, type of participants, type of intervention, type of comparator, and type of outcomes measured. Differences between the two review authors were resolved by discussion and consensus.

### 2.5. Assessment of Risk of Bias in Included Studies

Two review authors (EM and CI) independently assessed the risk of bias within each included study by addressing seven specific domains, namely, random sequence generation, allocation concealment, blinding of participants and personnel, blinding of outcome assessment, incomplete outcome data, selective outcome reporting, and “other issues” [21]. For each included study, the two review authors independently described what the study authors reported that they did for each domain and then made a decision relating to the risk of bias for that domain by assigning a judgement of “low risk” of bias, “high risk” of bias, or “unclear risk” of bias. The review authors compared the results of their independent assessments of risk of bias and resolved any discrepancies by discussion and consensus.

## 3. Results

### 3.1. Results of the Search

**Figure 1:**
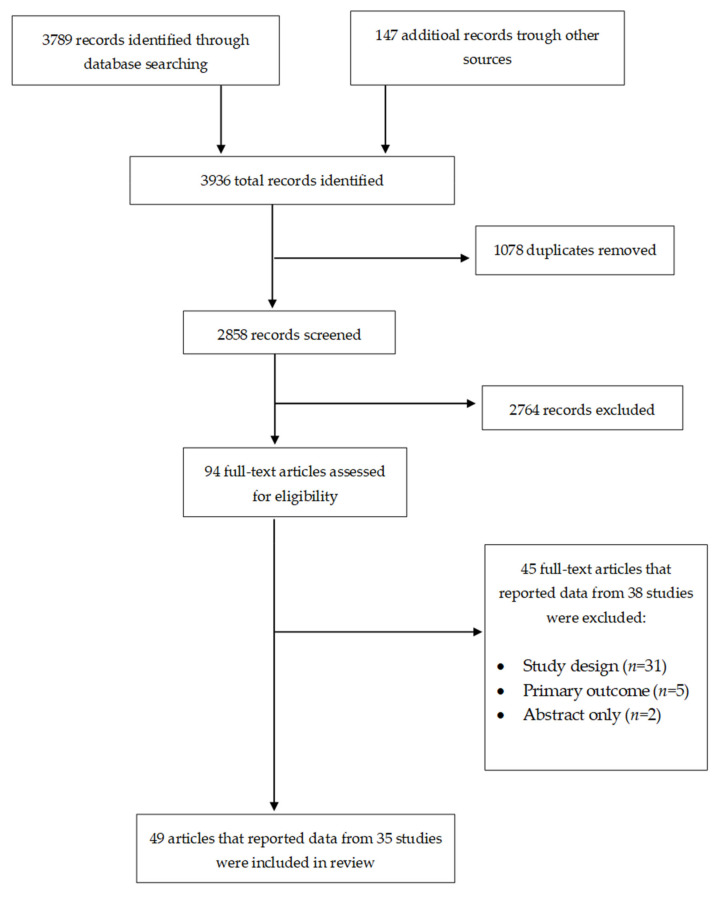
PRISMA flow diagram showing the study search and selection process.

The search yielded 3936 records. After removing 1078 duplicates, 2858 titles and abstracts were screened, and 2764 were not relevant. We reviewed the remaining 94 potentially eligible full-text articles for inclusion; 49 met our inclusion criteria, and we excluded 45 articles. The 49 included publications reported data on 35 studies. The 45 excluded articles reported data on 38 studies. The process used for the search and selection of studies for this review is described in Figure 1.

### 3.2. Description of Studies

The characteristics of the included studies are summarized in Appendix A, Table A2.

#### 3.2.1. Study Design and Setting

Thirty-two studies were randomized trials [22,23,24,25,26,27,28,29,30,31,32,33,34,35,36,37,38,39,40,41,42,43,44,45,46,47,48,49,50,51,52,53], two studies were controlled before–after studies [54,55], and one study was a non-randomized trial [56]. Thirty-two studies were conducted in the USA [22,23,24,25,26,27,28,29,30,31,32,33,34,35,37,39,40,41,42,43,44,45,46,47,48,50,51,52,53,54,55,56]. The remaining three studies were carried out in the UK [36], the Netherlands [38], and Australia [49].

#### 3.2.2. Participants

Seven studies were conducted among females only [22,24,27,33,36,37,51]; one study was conducted among males only [41]; one study was conducted among males and females [43]; thirteen studies were conducted among parents/guardians [25,31,32,34,38,39,40,42,44,45,47,49,56]; nine studies were conducted among providers [23,28,29,30,46,52,53,54,55]. The remaining four studies were conducted among mixed participants: adolescents and parents/guardians [35,48,50] and young adults and parents/guardians [26].

#### 3.2.3. Interventions and Comparators

Twenty-six studies assessed recipient-oriented interventions [22,24,25,26,27,31,32,33,34,35,36,37,38,39,40,41,42,43,44,45,47,48,49,50,51,56]. The remaining nine studies assessed provider-oriented interventions [23,28,29,30,46,52,53,54,55]. Comparators ranged from the standard of care in each setting to alternative interventions.

#### 3.2.4. Outcome Measures

All included studies reported data on our primary outcome, HPV vaccination coverage. Twenty-two studies reported data on the initiation of the HPV vaccine series [22,23,25,27,28,29,30,31,32,33,34,35,36,37,39,41,44,48,53,54,55,56]. Nineteen studies reported data on the completion of HPV vaccine series [22,24,25,27,30,34,36,40,41,42,43,44,46,48,51,52,54,55,56]. Four studies reported data on the receipt of any HPV vaccine dose [26,38,49,50].

Only four studies reported data on our pre-specified secondary outcomes. Three studies reported data on the cost of the intervention strategies [25,45,47], and one study reported data on adverse effects of the intervention [44].

#### 3.2.5. Excluded Studies 

Thirty-eight studies were excluded for reasons described in the characteristics of excluded studies (Appendix A, Table A3).

#### 3.2.6. Risk of Bias in Included Studies

The risk of bias in the included studies is summarized in Appendix A, Table A4. Below, we briefly describe the risk related to sequence generation, allocation concealment, blinding, completeness of outcome data, selective reporting, and other potential biases.

The risk of bias linked to the adequacy of the generation of the randomization sequence was low for twenty-two studies [22,23,25,26,27,28,32,33,34,36,37,39,40,41,44,45,46,47,48,49,51,52], unclear for ten studies [24,29,30,31,35,38,42,43,50,53], and high for two studies [55,56].

The risk of bias resulting from the adequacy of allocation concealment was low for five studies [22,27,37,46,47], unclear for fourteen studies [23,24,26,28,29,30,31,35,38,41,42,43,50,53], and high for fifteen studies [25,32,33,34,36,39,40,44,45,48,49,51,52,55,56].

The risk of bias linked to the adequacy of blinding of participants and research personnel was low for thirteen studies [22,26,32,39,42,43,45,47,48,49,51,52,53], unclear for thirteen studies [24,25,30,31,33,35,36,37,40,46,50,55,56], and high for eight studies [23,27,28,29,34,38,41,44].

The risk of bias related to the blinding of outcome assessors was low for four studies [24,26,32,40], unclear for twenty-six studies [22,25,27,28,29,30,31,33,36,37,39,42,43,44,45,46,47,48,49,50,51,52,53,55,56], and high for four studies [23,34,38,41].

The risk of bias linked to the completeness of outcome data was low for twenty-five studies [23,24,27,28,29,30,31,32,33,34,35,36,37,40,41,43,44,45,46,48,49,50,51,52,53], unclear for three studies [25,47,55], and high for six studies [22,26,38,39,42,56].

We did not find evidence of reporting bias or other biases beyond the ones reported above. 

### 3.3. Effects of Interventions

#### 3.3.1. Recipient-Oriented Interventions

##### Comparison 1: Tailored Education Compared to Standard of Care

Three studies assessed the effect of HPV-tailored education compared to the standard of care on the initiation of the HPV vaccine series. The studies showed that HPV-tailored education had no effect on the initiation of the HPV vaccine series (RR 1.00, 95% CI 0.86 to 1.17; 1350 participants) [22,27,48]. We judged the certainty of the evidence as very low because of concerns regarding the risk of bias in the included studies and serious imprecision in the findings.

Three studies assessed the effect of HPV-tailored education compared to the standard of care on the completion of the HPV vaccine series. Meta-analysis of data from these three studies showed that tailored education improved the completion of HPV vaccination series (RR 1.35, 95% CI 1.03 to 1.77; I^2^ = 27%; 880 participants) [22,27,51]. We downgraded the certainty of the evidence to low because of study limitations (i.e., a high risk of bias in all studies).

Two studies assessed the impact of tailored education compared to the standard of care on receipt of any dose of the HPV vaccine. The study showed that tailored education had no effect on uptake of HPV vaccine (RR 1.01, 95% CI 0.98 to 1.04; 8931 participants) [26,38]. We judged the certainty of the evidence as very low because of concerns regarding the risk of bias in the included studies and serious imprecision in the findings.

The studies reported no relevant secondary outcomes.

##### Comparison 2: Tailored Education Compared to Untailored Education

One study assessed the effect of tailored education compared to untailored education on receipt of any dose of HPV vaccine. The study showed untailored education had a slight effect on uptake of HPV vaccine compared to the tailored education intervention (RR 0.97, 95% CI 0.80 to 1.19; 855 participants) [26]. We downgraded the certainty of the evidence to very low because of concerns regarding the risk of bias in the included study and serious imprecision in the findings.

The study reported no relevant secondary outcomes.

##### Comparison 3: Narrative Education Compared to Non-Narrative Education 

Two studies showed that narrative education improved the initiation of the HPV vaccination series compared to non-narrative education (RR 1.38, 95% CI 0.95 to 2.00; I^2^ = 24%; 728 participants) [33,35]. We judged the certainty of the evidence as very low because of concerns regarding the risk of bias in the included studies and very serious imprecision in the findings.

The studies reported no relevant secondary outcomes.

##### Comparison 4: Multicomponent Education Compared to Standard of Care

A study showed that a multicomponent HPV education led to a very small decrease in the uptake of HPV vaccine compared to the standard of care (RR 0.98, 95% CI 0.87 to 1.11; 2912 participants) [50]. We downgraded the certainty of the evidence to low because of concerns regarding the risk of bias in the included study and serious imprecision in the findings.

The study reported no relevant secondary outcomes.

##### Comparison 5: Outreach Plus Reminders Compared to Standard of Care

One study assessed the impact of outreach plus reminders compared to the standard of care on the initiation of the HPV vaccine series. The study showed that the intervention improved the initiation of the HPV vaccine series (RR 1.28, 95% CI 1.02 to 1.60; 1624 participants) [31]. We judged the certainty of the evidence as moderate because of an unclear risk of bias in the included study.

The study reported no relevant secondary outcomes.

##### Comparison 6: Outreach Plus Education Compared to Standard of Care

A study assessed the impact of education and outreach compared to the standard of care on the initiation of the HPV vaccine series. The study reported that 84% of participants in both groups (Brochure only and *Entre Madre e Hija* (EMH)) initiated HPV vaccination, and no differences were observed between EMH program and brochure-only participants [56]. We downgraded the certainty of the evidence to moderate because of study limitations (i.e., non-randomized study).

One study assessed the impact of education and outreach compared to standard of care on the completion of the HPV vaccine series. The study showed that the intervention improved the completion of the HPV vaccine series (RR 1.70, 95% CI 1.30 to 2.22; 288 participants) [56]. We downgraded the certainty of the evidence to moderate because of study limitations (i.e., non-randomized study).

The study reported no relevant secondary outcomes.

##### Comparison 7: Education Plus Reminders Compared to Standard of Care

A study assessed the effect of education plus reminders compared to the standard of care on the initiation of the HPV vaccine series. The study showed that the intervention improved the initiation of the HPV vaccine series (RR 1.74, 95% CI 1.10 to 2.76; 150 participants) [41]. We downgraded the certainty of the evidence to low because of study limitations, as the included study had a high risk of bias.

Another study assessed the impact of HPV education plus reminders compared to the standard of care on the initiation of the HPV vaccine series. The study showed that the intervention was significantly associated with HPV vaccine uptake (RR: 0.84; 95% CI: 0.31–2.28) [37]. We judged the certainty of the evidence as very low because of concerns regarding the risk of bias in the included study and serious imprecision in the findings. 

Three studies assessed the impact of HPV education plus reminders compared to the standard of care on the completion of the HPV vaccine series. A meta-analysis of data from these three studies showed that the intervention improved the completion of the HPV vaccine series (RR 1.18, 95% CI 0.92 to 1.51; I^2^ = 28%; 6711 participants) [41,42,43]. We downgraded the certainty of the evidence to very low because of concerns regarding the risk of bias in the included studies and serious imprecision in the findings.

The studies reported no relevant secondary outcomes.

##### Comparison 8: Reminders vs. Standard of Care

Three studies assessed the effect of a reminder compared to the standard of care on the initiation of the HPV vaccine series. Two studies showed that the intervention improved the initiation of the HPV vaccine series (RR 1.16, 95% CI 1.13 to 1.18; I^2^ = 40%; 166,264 participants) [25,39]. We judged the certainty of the evidence as low because of study limitations, as the included studies had a high risk of bias.

Suh (2012) [45] reported that 26.5% of female adolescents initiated HPV vaccine series in the intervention group compared to 15.3% in the control group. We judged the certainty of the evidence as low because of study limitations, as the included studies had a high risk of bias.

Four studies assessed the effect of reminders compared to the standard of care on the completion of the HPV vaccine series. The study showed that intervention improved the completion of the HPV vaccination series (RR 1.23, 95% CI 1.18 to 1.29; I^2^ = 63%; 175,743 participants) [24,25,40,48]. We downgraded the certainty of the evidence to very low because of concerns regarding the risk of bias and serious inconsistency in the included studies.

Tull (2019) [49] assessed the effect of reminders compared to the standard of care on the uptake of any HPV dose. The study found that the intervention had no effect on the uptake of HPV vaccine (RR 1.03, 95% CI 1.01 to 1.05; 5912 participants). We judged the certainty of the evidence as moderate because of a high risk of bias in the included study. 

Three studies measured the costs of the intervention [25,45,47]. Coley (2018) [25] calculated the reminder mailing and vaccination costs. The mailing costs were $13,698 for address verification, $44,312 for printing, and $57,991 for postage. The vaccination cost was $30.95 per adolescent who initiated the HPV vaccine series. Szilagyi (2013) [47] measured the cost of the intervention on pertussis, meningococcal, and HPV vaccination among adolescents. The delivery cost of the intervention was $18.78 for mailed and $16.68 for phone reminders per adolescent per year, respectively. The cost per additional fully vaccinated adolescent was $463.99 for mailed and $714.98 for telephone reminders. Suh (2012) [45] calculated the total operating cost of reminder/recall intervention per additional adolescent who received tetanus-diphtheria-acellular pertussis, meningococcal conjugate, or a first dose of human papillomavirus vaccine in four practices. The total operating cost, which included personnel and supply costs, ranged between $1087 and $1349.

##### Comparison 9: Educational Reminders Compared to Plain Reminders

Hofstetter (2017) [32] showed that educational reminders improve the initiation of the HPV vaccination series compared to plain reminders (RR 0.53, 95% CI 0.27 to 1.06; 90 participants). We downgraded the certainty of the evidence to very low because of concerns regarding the risk of bias in the included study and serious imprecision in the findings.

The study reported no relevant secondary outcomes.

##### Comparison 10: Financial Incentives Plus Reminders Compared to Standard of Care

One study assessed the impact of financial incentives plus reminders compared to the standard of care on the initiation of the HPV vaccine series. The study showed that intervention improved the initiation of the HPV vaccine series (RR 1.73, 95% CI 1.34 to 2.24; I^2^ = 64%; 1000 participants) [36]. We judged the certainty of the evidence as very low because of concerns regarding the risk of bias in the included study and serious inconsistency.

A study assessed the impact of financial incentives plus reminders compared to the standard of care on the completion of the HPV vaccine series. The study showed that intervention improved the initiation of the HPV vaccine series (RR 1.82, 95% CI 1.26 to 2.63; I^2^ = 0%; 1000 participants) [36]. We downgraded the certainty of the evidence to low because of a high risk of bias in the included study.

The study reported no relevant secondary outcomes.

##### Comparison 11: Brief Motivational Behavioral Intervention Compared to Standard of Care

One study assessed the impact of the brief motivational behavioral intervention compared to the standard of care on the initiation of the HPV vaccine series. The study showed that intervention improved initiation of the HPV vaccine series (RR 1.10, 95% CI 0.85 to 1.43; 200 participants) [34]. We downgraded the certainty of the evidence to very low because of concerns regarding the risk of bias in the included study and serious imprecision in the findings.

A study assessed the impact of the brief motivational behavioral intervention compared to the standard of care on the completion of the HPV vaccine series. The study showed that intervention improved the completion of HPV vaccine series (RR 1.73, 95% CI 0.66 to 4.59; 200 participants) [34]. We judged the certainty of the evidence as very low, because of concerns regarding the risk of bias in the included studies and serious imprecision in the findings.

The study reported no relevant secondary outcomes.

##### Comparison 12: Brief Health Messaging Using Different Formats

One study assessed the effect of brief health messaging on the initiation of the HPV vaccine series. The study reported that rhetorical questions did not increase the initiation of the HPV vaccine series (RR = 1.15, CI 0.89, 1.50). One-sided and two-sided messages also had no effect on the initiation of the HPV vaccine series [44]. We downgraded the certainty of the evidence to very low because of concerns regarding the risk of bias in the included study and serious imprecision in the findings.

A study assessed the effect of brief health messaging on the completion of the HPV vaccine series. The study reported that rhetorical questions and message sidedness had no significant effect on the completion of the HPV vaccine series [44]. We judged the certainty of the evidence as very low because of concerns regarding the risk of bias in the included studies and serious imprecision in the findings.

Rickert (2015) evaluated the adverse events of the intervention, but none occurred. 

#### 3.3.2. Provider-Oriented Intervention 

##### Comparison 13: Prompts Compared to Standard of Car

One study assessed the impact of provider prompts compared to the standard of care on the initiation of the HPV vaccine series. The study showed that provider prompts improved the initiation of the HPV vaccine series (RR 1.36, 95% CI 1.20 to 1.54; 925 participants) [53]. We downgraded the certainty of the evidence to moderate because of study limitations, as the included study had an unclear risk of bias. 

Two studies assessed the effect of provider prompts compared to the standard of care on the completion of the HPV vaccine series. The study showed that intervention improved the completion of the HPV vaccine series (RR 1.12, 95% CI 1.06 to 1.19; I^2^ = 72%; 3056 participants) [46,52]. We downgraded the certainty of the evidence to very low because of concerns regarding the risk of bias in the included studies and serious inconsistency. 

The studies reported no relevant secondary outcomes. 

##### Comparison 14: Provider Training Compared to Standard of Care

A study assessed the effect of provider announcement and conversation training compared to the standard of care on the initiation of the HPV vaccine series. The study reported that clinics that received announcement training had increases in HPV vaccine initiation coverage that exceeded control clinics’ increases (5.4% difference, 95% CI 1.1 to 9.7). Clinics that received conversation training did not differ from the control arm on uptake for HPV vaccine initiation (all *P*s > 0.05) [23]. We judged the certainty of the evidence as very low because of concerns regarding the risk of bias in the included studies and serious imprecision in the findings.

The study reported no relevant secondary outcomes. 

##### Comparison 15: Provider Training Plus Assessment and Feedback Compared to Wait List Control

One study assessed the impact of provider training plus assessment and feedback intervention compared to wait list control on the initiation of the HPV vaccine series. The study showed that initiation of the HPV vaccine series rates increased by 10.2 percentage points in the intervention arm and 6.9 percentage points in the control arm [29]. We downgraded the certainty of the evidence to very low because of concerns regarding the risk of bias in the included studies and very serious imprecision in the findings.

The study reported no relevant secondary outcomes.

##### Comparison 16: Assessment and Feedback Compared to Standard of Care Series

Irving (2018) [54] evaluated the effect of assessment and feedback intervention compared to the standard of care on the initiation of the HPV vaccine series among adolescent boys and girls aged 11–17 years. The study reported that there was no significant difference in the initiation of the HPV vaccine series between intervention and control clinics. We downgraded the certainty of the evidence to very low because of study limitations (i.e., before–after study).

One study evaluated the effect of assessment and feedback intervention compared to the standard of care on the initiation of the HPV vaccine series among adolescent boys and girls aged 11–17 years [54]. The study found that the completion of the HPV vaccine series between the intervention and control clinics was not significantly different. We downgraded the certainty of the evidence to very low because of study limitations (i.e., before–after study).

The study reported no relevant secondary outcomes.

##### Comparison 17: Provider Consultation Compared to Standard of Care

A study assessed the effect of in-person and webinar-delivered Assessment, Feedback, Incentives, and eXchange (AFIX) consultations compared to standard of care on the initiation of the HPV vaccine series. The study reported that participants served by clinics in the in-person arm had uptake that exceed those in the control arm for HPV vaccine initiation (1.5% (95% CI: 0.3 to 2.7)). Participants served by clinics in the webinar versus control arms also had larger coverage increases for HPV vaccine initiation (1.9 (95% CI: 0.7 to 3.1)) [28]. We downgraded the certainty of the evidence to very low because of concerns regarding the risk of bias in the included study and very serious imprecision in the findings. 

The study reported no relevant secondary outcomes.

##### Comparison 18: Funding Compared to Training and Technical Assistance

One study compared the effect of $90,000 (2-year grant fund), $10,000 (3-month grant fund), and training and technical assistance on the initiation of the HPV vaccine series among patients aged 11–12 years. The study found that initiation of the HPV vaccine series rates increased by 18.4, 14.6, and 11.1 percentage points in the $90,000 grant fund, training and technical assistance, and $10,000 grant fund, respectively [30]. We judged the certainty of the evidence as low because of concerns regarding the risk of bias in the included study and serious imprecision in the findings. 

A study compared the effect of $90,000 (2-year grant fund), $10,000 (3-month grant fund), and training and technical assistance on the completion of the HPV vaccine series among patients aged 11–12 years. The study reported that completion of HPV vaccine series rates increased only in the $90,000 grant fund by 5 percentage points and decreased by 4.5 and 1.7 percentage points in the $10,000 grant fund and training and technical assistance arm, respectively [30]. We judged the certainty of the evidence as low because of concerns regarding the risk of bias in the included study and serious imprecision in the findings. 

The study reported no relevant secondary outcomes. 

##### Comparison 19: Multicomponent Intervention Compared Standard of Care

One study assessed the impact of a multicomponent intervention compared to the standard of care on the initiation of the HPV vaccine series among adolescents aged 11–12 and 13–17 years. Among adolescents aged 11–12 years, HPV vaccine series initiation rates increased by 18.7 percentage points in the intervention arm and 12.6 percentage points in the control arm, whereas, among adolescents aged 13–17 years, the rates increased by 8.7 percentage points in the intervention arm and 7 percentage points in the control arm [55]. We downgraded the certainty of the evidence to very low because of study limitations (i.e., before–after study).

A study assessed the impact of a multicomponent intervention compared to the standard of care on the completion of the HPV vaccine series among adolescents aged 11–12 and 13–17 years. HPV vaccine series completion rates among adolescents aged 11–12 years increased by the same 20.7 percentage points both in the intervention and control arms, whereas, among adolescents aged 13–17 years, the completion rates increased by 12.5 percentage points in the intervention and 11.9 percentage points in the control arms [55]. We downgraded the certainty of the evidence to very low because of study limitations (i.e., before–after study).

The study reported no relevant secondary outcomes.

## 4. Discussion

Our study found that recipient-oriented interventions that improved the initiation of the HPV vaccine series were narrative education, reminders, outreach plus reminders, education plus reminders, financial incentives plus reminders, and brief motivational behavioral interventions. We also found that the recipient-oriented interventions that improved the completion of the HPV vaccine series were tailored education, outreach and education, education plus reminders, reminders in general, financial incentives plus reminders, and brief motivational behavioral interventions. Tailored education, outreach and education, and brief health messaging were recipient-oriented interventions that had no effect on the initiation of the HPV vaccine series. Brief health messaging was also found to be a recipient-oriented intervention that had no effect on the completion of the HPV vaccine series. The provider-oriented interventions that improved the initiation of the HPV vaccine series were prompts, training, training plus assessment and feedback, consultation, funding**,** and multicomponent interventions. Prompts, funding and multicomponent were also found to be provider-oriented interventions that improved the completion of HPV vaccine series. Assessment and feedback were provider-oriented interventions that had no effect on both the initiation and the completion of the HPV vaccine series. With regards to the improvement of uptake of any HPV vaccine dose, all assessed recipient-oriented interventions, tailored education, untailored education, multicomponent education, and reminders did not have any effect.

Our systematic review was comprehensive. We included all known types of interventions, including recipient- and provider-oriented interventions, and all country settings. Our comprehensive search resulted in 35 studies that met our inclusion criteria. However, all studies were conducted in high-income countries, mainly the USA, where the burden of HPV is relatively low. None of the included studies were conducted in low-income countries, where the burden of HPV is very high. Therefore, the findings of these studies may be applicable only in the settings of the high-income countries. Another limitation is that there is very small number of studies that reported data on our secondary outcomes. Among the included studies, there were only one and three studies that reported data on the adverse effects and the cost of the interventions, respectively. However, because of variations in the measures of costs between the three studies, we were unable to conduct a meta-analysis. One study that reported on the adverse effects of the intervention stated that there were no effects documented in the study. Given that there is insufficient data on adverse effects and costs of the interventions, there is an urgent need for more studies to address these gaps. In addition, these studies should be well-designed and should evaluate outcomes and report results in ways that will allow the clear assessment of the cost and adverse effects of the interventions.

Thirty-five studies were excluded in this review mainly based on the methods used to conduct them. In addition, most of these studies were published after 2015, the period in which a previous similar review by Smulian (2016) [17] included studies up to. We may therefore have missed important findings from these studies. Well-designed studies that assess the effect of the interventions on HPV vaccination coverage are needed.

We used the Grading of Recommendations, Assessment, Development and Evaluations (GRADE)approach to assess the certainty of the evidence on the effects of the included interventions on HPV vaccination coverage. Among the recipient-oriented interventions that improved HPV vaccination coverage, we judged the certainty of the evidence as moderate for outreach plus reminders, low for reminders, and very low for education, financial incentives plus reminders, and brief motivational behavioral interventions. Regarding provider-oriented interventions that improved HPV vaccination coverage, we judged the certainty of the evidence as moderate for provider prompts, low for funding, and very low for training, consultation, training plus assessment and feedback, consultation, and multicomponent interventions. Overall, the certainty of evidence of interventions that improved HPV vaccination coverage was very low to moderate. Our main concerns with the evidence related to study limitations: risk of bias, indirectness, and imprecision in the studies. There is, therefore, an urgent need for well-designed, well-implemented, and well-reported studies to increase the certainty of the current evidence. We minimized potential biases in the review process by adhering to the Cochrane guidelines for conducting a systematic review [21]. We conducted comprehensive searches of both peer-reviewed and grey literature, without limiting the searches to a specific language. Two review authors independently assessed study eligibility, extracted data, and assessed the risk of bias in each included study. We are not aware of any biases in the review process.

Several systematic reviews have assessed the effectiveness of interventions for improving HPV vaccination coverage [6,13,14,15,16,17,57]. Smulian (2016) [17] evaluated the effectiveness of the interventions for improving HPV vaccination coverage in USA. The review found that many types of intervention strategies (targeting recipients, providers, and the health system) increased HPV vaccination coverage in different settings. Contrary to our review, which included 35 studies, this similar comprehensive review, which searched five databases for studies published between 2006 to 2015, resulted in 34 eligible studies. Like their review, all the studies included in our review were conducted in high-income countries. Of the 35 studies included in our review, 32 were conducted in the USA and the remaining three were from Australia, the Netherlands, and the UK. Acampora (2020) [13] and colleagues evaluated the effectiveness of interventions for improving HPV vaccination coverage among adolescents. The authors found that reminder-based interventions, either alone or in combination with other interventions, had a positive effect on vaccination coverage [13]. In another review, the effectiveness of intervention for improving HPV coverage among college students was assessed. The authors reported that the educational intervention that utilized a joint peer and medical provider message was the only intervention in their review that significantly increased HPV vaccine uptake [14]. The effectiveness of communication technology interventions on HPV vaccination coverage was assessed by Francis (2017) [16] and found that usage of computer, mobile, or internet technologies as the sole or primary mode for intervention delivery increased vaccination coverage. Niccolai (2015) [6] conducted a systematic review to assess the effectiveness of practice- and community-based interventions on improving HPV vaccination coverage. The review reported that several interventions including reminder and recall systems, physician-focused strategies (e.g., audit and feedback), school-located programs, and social marketing have improved vaccination coverage. The effectiveness of the interventions that applied new media to improve vaccination coverage was assessed by Odone and colleagues. The authors reported that text messaging, accessing immunization campaign websites, using patient-held web-based portals and computerized reminders, and standing orders increased vaccination coverage rates [57]. Walling and colleagues compared the effectiveness of the informational-, behavioral-, and environmental-based interventions on improving HPV vaccination coverage among adolescents and young adults aged 11 to 26 years. The authors found that environmental interventions, particularly school-based vaccination programs were most effective in increasing vaccination coverage [15].

## 5. Conclusions

Although several interventions improved HPV vaccination coverage, the certainty of the evidence varied from moderate to low. Although many studies were included in our review, all of them were conducted in high-income countries. There is, therefore, a need for further high-quality studies in low- and middle-income countries. At the same time, many studies assessing the effect of different interventions on improving HPV vaccination coverage were excluded because of the way they were conducted. As a result, well-designed, well-implemented, and well-reported studies are needed. In addition, given that there is limited information from existing studies on the cost of the tested interventions, further studies are needed to address this challenge.

## Data Availability

Not applicable.

## Appendix A

**Table A1:** Search strategies (search date: 9 July 2019).

Search	Query	Results
PubMed		
**#1**	Search (“papillomavirus vaccines”(MeSH Terms) OR (“papillomavirus”[All Fields] AND “vaccines”[All Fields]) OR “papillomavirus vaccines”(All Fields) OR (“hpv”[All Fields] AND “vaccine”[All Fields]) OR “hpv vaccine”(All Fields)) AND (VACCINATE[All Fields] OR [“vaccination”[MeSH Terms] OR “vaccination”[All Fields]))	6876
**#2**	Search (randomized controlled trial(pt) OR controlled clinical trial(pt) OR randomized(tiab) OR placebo(tiab) OR “drug therapy”(Subheading) OR randomly(tiab) OR trial(tiab) OR groups(tiab)) NOT (“animals”(MeSH Terms) NOT “humans”(MeSH Terms))	3,933,624
**#3**	Search (“case-control studies”(MeSH Terms) OR (“case-control”[All Fields] AND “studies”[All Fields]) OR “case-control studies”(All Fields) OR (“case”[All Fields] AND “control”[All Fields] AND “studies”[All Fields]) OR “case control studies”(All Fields)) OR (“cohort studies”[MeSH Terms] OR [“cohort”[All Fields] AND “studies”[All Fields]] OR “cohort studies”(All Fields))	2,188,056
**#4**	Search (#2 OR #3)	5,407,771
**#5**	Search (#1 AND #4)	1815
**Web of Science**		
**#1**	Search ((“papillomavirus vaccines” OR [“papillomavirus” AND “vaccines”] OR “papillomavirus vaccines” OR [“hpv” AND “vaccine”] OR “hpv vaccine”) AND (VACCINATE OR [“vaccination” OR “vaccination”]))	5810
**#2**	Search (([randomized controlled trial] OR [controlled clinical trial]) OR ([“case-control studies” OR [“case-control” AND “studies”] OR [“case”AND “control” AND “studies”] OR “case control studies”] OR [“cohort studies” OR [“cohort” AND “studies”]]))	652,297
**#3**	Search (#2 AND #1)	669
**Scopus**		
**#1**	Search (“papillomavirus vaccines” OR “papillomavirus vaccine” OR “hpv vaccine” OR “HPV vaccines”)	9447
**#2**	Search (“Randomized controlled trial” OR “controlled clinical trial” OR “Randomized Controlled trials” OR “Controlled Clinical trials” OR “case-control studies” OR “Case control studies”)	1,175,572
**#3**	Search (#1 AND #2)	738

**Table A2:** Characteristics of included studies.

No.	Study Id	Country	Study Type	Sample Size	Participants	Intervention	Comparator	Outcome Measure
1	Bennett (2015) [22] Bennett (2014) [58] NCT01769560 [59]	USA	RCT	661	Female students aged 18–26 years	330 participants were randomized to individually tailored educational website.	331 participants were randomized to the website of the standard CDC information factsheet on the HPV vaccine.	Initiation and completion of HPV vaccine series
2	Brewer (2017) [23] NCT02377843 [60]	USA	RCT	30	Providers	10 clinics were randomized to announcement training. Participating clinicians received 1 h of training on announcement to recommend HPV vaccination. 10 clinics were randomized to conservation training. Participating clinicians received 1 h of training on conservation to recommend HPV vaccination.	10 clinics were randomized to the waitlist control condition. Participating clinics received a video recording of the announcement training, which was sent 1 month after the 6-month assessment of vaccination outcomes.	Initiation of HPV vaccine series
3	Chao (2015) [24]	USA	RCT	12,225	Females aged 9–26 years	9804 participants were randomized to reminder letter. Participants received a letter reminding them of the HPV vaccination.	2451 participants were randomized to the standard of care. Participants received no reminder letters.	Completion of HPV vaccine series
4	Coley (2018) [25]	USA	RCT	303,965	Parents of adolescents aged 11–13 years	151,982 participants were randomized to reminder letter. Parents received letters reminding them to vaccinate their adolescents.	151,983 participants were randomized to control letters. Participants received letters six months after the observation period was completed.	Initiation and completion of HPV vaccine series Cost of intervention
5	Dempsey (2019) [26] NCT02145156 [61]	USA	RCT	1294	Young adults aged 18–26 years and their parents	430 participants were randomized to web-based tailored messaging called CHICOs (Combatting HPV Infections and Cancers). Participants received an iPad with the CHICOS intervention programmed onto it. 425 participants were randomized to web-based untailored messaging. Participants received an iPad-based version of the Vaccine Information Sheet from the Centers for Disease Control and Prevention.	439 participants were randomized to usual care. Participants received care routinely provided by the clinician and did not interact with or have access to the iPad	Receipt of any HPV vaccine dose
6	DiClemente (2015) [27] NCT00813319 [62]	USA	RCT	216	Female adolescents aged 14–18 years	108 participants were randomized to theory-based, multi-component computer-delivered media-based intervention called Girls OnGuard. Participants viewed a 12-min interactive computer-delivered media presentation on HPV vaccination.	108 participants were randomized to placebo. Participants viewed a time-equivalent health promotion media presentation on physical activity and nutrition.	Initiation and completion of HPV vaccine series
7	Fisher-Borne (2018) [30]	USA	RCT	30	Providers	10 participants were randomized to $90,000 2-year grant. 10 participants were randomized to $10,000 3-month grant.	10 participants were randomized to no funding. Participants received training and technical assistance.	Initiation and completion of HPV vaccine series
8	Gilkey (2014) [28]	USA	RCT	91	Providers Primary care clinics (pediatric and family practice clinics) serving adolescents 11–18 years old.	30 clinics were randomized to in-person delivered Assessment, Feedback, Incentives, and eXchange (AFIX) consultation. 30 clinics were randomized to webinar-delivered AFIX consultation.	30 clinics were randomized to no consultation	Initiation of HPV vaccine series
9	Gilkey (2019) [29]	USA	RCT	78	Pediatricians	43 participants were randomized to quality improvement plus assessment and feedback.	35 participants were randomized to wait-list control arm. Participants received QI program after 6 months of follow-up.	Initiation of the HPV vaccine series
10	Henrikson (2018) [31] Henrikson (2017) [63]	USA	RCT	1805	Parents of adolescents aged 10–12 years	1354 participants were randomized to outreach letter, brochure, and reminder. Participants received outreach letter and brochure recommending HPV vaccination followed by automated HPV vaccine reminder call for dose 1.	451 participants were randomized to usual care. Participants received no outreach letter or reminder call.	Initiation of the HPV vaccine series
11	Hofstetter (2017) [32]	USA	RCT	295	Parents of adolescents with chronic medical conditions	154 participants were randomized to educational text message reminders. Participants received educational text message reminders on receipt of HPV.	141 participants were randomized to plaint text message reminders.	Initiation of the HPV vaccine series
12	Hopfer (2012) [33]	USA	RCT	404	College women aged 18–26 years	252 participants were randomized to narrative messages Participants viewed one of three videos: (1) a video of vaccine decision narratives delivered by peers (101), (2) a video of narratives delivered by medical experts (50), or (3) a video of narratives delivered by a combination of peers and experts (101)	152 participants were randomized to no narrative messages Participants viewed one of three controls: (1) an informational video without narratives, (2) the campus website providing information about HPV and the vaccine, or (3) no message.	Initiation of the HPV vaccine series
13	Irving (2018) [54]	USA	BA	12	Providers (clinics)	9 clinics were enrolled in the provider-focused assessment and feedback intervention.	3 clinics were enrolled in the standard of care.	Initiation and completion of HPV vaccine series
14	Joseph (2016) [34] NCT01254669 [64]	USA	RCT	200	Mothers of daughters aged 11–15 years	100 participants were randomized to brief negotiated interviewing (BNI).Participants received the BNI intervention, which addressed mothers’ beliefs, values, and concerns about HPV prevention and accounting for their priorities for health and well-being.	100 participants were randomized to no BNI. Participants received the low literacy, standard-practice HPV vaccine information sheet given to all patients prior to vaccination	Initiation and completion of HPV vaccine series
15	Lee (2018) [35]	USA	RC	19	Mothers and daughters aged 14–17 years dyads	10 participants were randomized to storytelling narrative videos. The participants watched a 26-min storytelling narrative DVD on HPV vaccine, entitled “Save My Daughter from Cervical Cancer.”	9 participants were randomized to written non-narrative education materials. Participants received CDC flyers on the HPV vaccine.	Initiation of the HPV vaccine series
16	Mantzari (2015) [36]	UK	RCT	1000	Girls aged 16–18 years	500 participants were randomized to financial incentives. Participants received the offer of “Love2Shop” vouchers worth £45 for receiving the three vaccinations.	500 participants were randomized to no financial incentives. Participants received no incentives.	Initiation and completion of HPV vaccine series
17	Mclean (2017) [55]	USA	BA	43	Providers (clinics)	9 participants were enrolled in the multi-component interventions. Participants received education on HPV vaccination, assessment and feedback, and patient reminder and recall notifications.	34 participants were enrolled in the standard of care.	Initiation and completion of HPV vaccine series
18	Parra Medina (2015) [56]	USA	N-RCT	372	Hispanic mothers with a daughter aged 11–17 years	257 participants were enrolled in the outreach and education program called Entre Madre e Hija (EMH), a culturally relevant cervical cancer prevention program. Participants received health education, referral, and navigation support for HPV vaccination. They also received an HPV vaccine educational brochure.	115 participants were enrolled in the HPV vaccine educational brochure only.	Initiation and completion of HPV vaccine series
19	Patel (2012) [37]	USA	RCT	256	Female college students aged 18-26 years	128 were randomized to HPV-specific patient education and reminder letter. Participants received HPV and Vaccination” fact sheet plus reminder letter for HPV vaccination.	128 were randomized to standard of care. Participants did not receive “HPV and Vaccination” fact sheet and reminder letter.	Initiation of the HPV vaccine series
20	Pot (2017) [38]	The Netherlands	RCT	806	Mothers of girls aged 12 years	3995 participants were randomized to web-based tailored intervention with virtual assistants. Participants received tailored information on HPV and HPV vaccination.	4067 participants were randomized to standard of care. Participants received universal information about the HPV vaccination	Receipt of any HPV vaccine dose
21	Rand (2015) [39]	USA	RCT	3812	Parents of adolescents aged 11–16 years	1893 participants were randomized to text message reminders. Parents received text message reminding them that their adolescents were due for HPV vaccine doses.	1919 participants were randomized to general adolescent health text message. Parents received general adolescent health text message each time their adolescents were due for HPV vaccine dose.	Initiation of the HPV vaccine series
22	Rand (2017) [40] NCT01731496 [65]	USA	RCT	749	Parents of adolescents aged 11–17 years	178 participants were randomized to telephone message reminder. Parents received telephone call reminding them that their adolescents were due for an HPV vaccine dose.191 participants were randomized to text message reminders. Parents received text message reminding them that their adolescents were due for HPV vaccine dose.	180 participants were randomized to standard of care (telephone reminder control). 200 participants were randomized to standard of care (text reminder control).	Completion of HPV vaccine series
23	Reiter (2018) [41] Mcree (2018) [66], NCT01769560 [59]	USA	RCT	150	Young gay and bisexual men aged 18–25 years	76 participants were randomized to outsmart HPV intervention. Participants received population-targeted, individually tailored content about HPV and the HPV vaccine, and monthly HPV vaccination reminders sent via email and/or text message.	74 participants were randomized to standard HPV information. Participants received standard information about HPV and the HPV vaccine.	Completion of HPV vaccine series
24	Richman (2019) [42]	USA	RCT	257	Parents of adolescences aged 9–17 years.	129 participants were randomized to electronic messaging (text or email).Participants received appointment reminders and education messages about HPV and the HPV vaccine.	128 participants were randomized to standard of care. Participants received a paper card with the date of their next appointment written on it.	Completion of HPV vaccine series
25	Richman (2016) [43]	US	RCT	264	College students aged 18–26 years	130 participants were randomized to electronic messaging (text or email). Participants received appointment reminders and education messages about HPV and the HPV vaccine. In addition, participants received a paper card with the date of their nextappointment written on it.	134 participants were randomized to standard of care. Participants received a paper card with the date of their next appointment written on it.	Completion of HPV vaccine series
26	Rickert (2015) [44]	USA	RCT	445	Parents of male and female adolescents aged 11–15 years	109 participants were randomized to rhetorical questions (RQ) plus one-sided message. 114 participants were randomized to RQ plus two-sided message.	116 participants were randomized to no RQ plus one-sided message. 106 participants were randomized to no RQ plus two-sided message.	Initiation and completion of HPV vaccine series
27	Suh (2012) [45]	USA	RCT	1600	Parents of adolescents aged 11 to 18 years	800 participants were randomized to letter and telephone reminders. Parents received letter and autodialed telephone call informing them that their adolescents were due for an HPV vaccination.	800 participants were randomized to usual care. Parents received no reminder/recall	Initiation and completion of HPV vaccine series Cost of intervention
28	Szilagyi (2015) [46]	USA	RCT	22	Providers / Primary care practices attendant by adolescents aged 11–17 years	11 practices were randomized to provider prompts on HPV vaccination (electronic health record (EHR) or nurse- or staff-initiated prompts).Participants received prompts indicating the specific HPV vaccine doses that the adolescents were due for during their practice visits.	11 practices were randomized to standard of care. Participants did not receive any prompts.	Completion of HPV vaccine series
29	Szilagyi (2013) [47]	USA	RCT	7404	Parents of adolescents aged 11–17 years	2494 participants were randomized to letter reminder. Parents received reminder letters advising them to call their adolescent’s primary care practice to schedule an appointment for HPV vaccination. 2504 participants were randomized to telephone reminder. Parents received autodialed reminder calls advising them to call their adolescent’s primary care practice to schedule an appointment for HPV vaccination.	2406 participants were randomized to standard of care. Parents received no reminder.	Initiation and completion of HPV vaccine series Costs of the intervention
30	Tiro (2015) [48]	USA	RCT	814	Parents and girls/daughters aged 11–18 years dyads	410 participants were randomized to HPV-vaccine-specific brochure and recalls. Participants received HPV-vaccine-specific brochures and telephone recalls for vaccination.	404 participants were randomized to general adolescent vaccine brochure. Participants received a CDC brochure about all Advisory Committee on Immunization Practices’ recommended vaccines.	Initiation and completion of HPV vaccine series
31	Tull (2019) [49]	Australia	RCT	4386	Parents of Year 7 students	1442 participants were randomized to motivational short message service (SMS) Reminders. Participants received a motivational SMS: “Vaccine preventable diseases are still a problem in the community and children most at risk are those that have not been immunized.” 1418 participants were randomized to self-regulatory SMS reminders. Participants received an SMS: “make a plan now for how your child will get to school on-time on immunization day.”	1526 participants were randomized to no SMS reminders. Participants received no SMS reminders.	Receipt of any HPV vaccine dose
32	Underwood (2019) [50] Herbert (2014) [67]	USA	RCT	2135	Parents and adolescents	668 participants (parents only) were randomized to educational intervention. Participants received an educational brochure about adolescent vaccines. 690 participants (parents and adolescents) were randomized to multicomponent educational intervention. Participants (parents) received educational brochures about vaccines recommended during adolescence. Participants (adolescents) received a vaccine-focused curriculum delivered by science teachers.	777 participants were randomized to no intervention. Parents received no information.	Receipt of any HPV vaccine dose
33	Vanderpool (2013) [51]	USA	RCT	344	Young women aged 18–26 years	178 participants were randomized to an educational DVD, entitled “1-2-3 Pap.” Participants watched a 13-min educational DVD on HPV, HPV vaccines, and pap tests	166 participants were randomized to Standard of care.	Completion of HPV vaccine series
34	Wilkinson (2019) [52] Zimet (2016) [68] NCT02558803 [69]	USA	RCT	29	Providers (pediatric clinicians)	15 participants were randomized to automated reminder. Participants received automated reminders via Child Health Improvement through Computer Automation (CHICA) to recommend the 2nd and 3rd doses of HPV vaccine to adolescents aged 11–17 years who had already initiated the vaccine series.	14 participants were randomized usual practice. Participants received reminders to recommend the 2nd and 3rd doses of HPV vaccine manually from the nurses who looked them up in the Children and Hoosier Immunization Registry Program (CHIRP).	Completion of HPV vaccine series
35	Zimet (2018) [53]	USA	RCT	29	Providers (health care providers)	8 participants were randomized to simple reminder prompt. Participants received computer-generated messages reminding them of HPV vaccination eligibility. 11 participants were randomized to elaborated reminder prompt. Participants received computer-generated reminders with a suggested script for recommending the three adolescent platform vaccines.	10 participants were randomized to usual practice. Participants did not receive any reminder prompt. They made HPV vaccination recommendations their existing methods for determining eligibility.	Initiation of the HPV vaccine series

**Table A3:** Characteristics of excluded studies.

Study No.	Study Id.	Reason
1	Chigbu (2017) [70]	A before–after study evaluating the impact of trained community health educators on the uptake of cervical and breast cancer screening and HPV vaccination. The study was excluded because it had one intervention and control group.
2	Cory (2019) [71]	A randomized study assessing the effects of educational interventions on human papillomavirus vaccine acceptability. Reported outcome was intention to vaccinate.
3	Daley (2014) [72]	A cluster-randomized controlled study assessing the program costs, the proportion of costs reimbursed, and the likelihood of vaccination in a school-located adolescent vaccination program that billed health insurance. One intervention and control cluster.
4	Davies (2017) [73] Skinner (2015) [74]	A cluster-randomized controlled study evaluating the effect of educational intervention on HPV vaccination uptake. One intervention and control cluster.
5	Dempsey (2018) [75] O’Leary (2017) [76] NCT02456077 [77]	A cluster-randomized controlled study evaluating the effect of a health care professional communication training intervention on adolescent human papillomavirus vaccination. One intervention and control cluster.
6	Deshmukh (2018) [78]	A before–after study evaluating the impact of a clinical intervention bundle on the rate of missed opportunities and uptake of the vaccine among young adult women. One intervention and control group.
7	Dixon (2019) [79] Dixon (2016) [80] NCT02546752 [81]	A cluster-randomized controlled study assessing the effects an educational intervention in improving HPV vaccination. One intervention and control cluster.
8	Fiks (2013) [82]	A cluster-randomized controlled study evaluating the effectiveness of decision support for families, clinicians, or both on HPV vaccine receipt. One intervention and control cluster.
9	Fiks (2016) [83]	A before–after study evaluating the impact of Maintenance-of-Certification program on improving HPV vaccination rates. One intervention and control group.
10	Forster (2017) [84]	A cluster-randomized controlled study evaluating the effect of an adolescent incentive intervention on improving HPV vaccination uptake. One intervention and control cluster.
11	Grandahl (2016) [85]	A cluster-randomized controlled study assessing the effect of the educational intervention on increasing HPV vaccination among adolescents. One intervention and control cluster.
12	Jacobs-Wingo (2017) [86]	A cross-sectional study assessing the impact of multi-component interventions on increasing HPV vaccine coverage.
13	Jiménez-Quiñones (2017) [87]	A descriptive study assessing the impact of a pharmacist administered educational program on the vaccination rates of HPV. A descriptive study.
14	Keeshin (2017) [88]	A prospective cohort study evaluating the impact of text message reminder recall on increasing HPV vaccination in young HIV-1-infected patients. A prospective study.
15	Kempe (2012) [89]	A demonstration study assessing the effectiveness and cost of immunization recall at school-based health centers. A demonstration study.
16	Kim (2018) [90]	Conference abstract only
17	Lee (2016) [91]	A before–after study evaluating the effect of the text messaging intervention on HPV vaccination among Korean-American women. One intervention group.
18	Mayne (2014) [92]	A cluster-randomized controlled study evaluating the effect of decision support on HPV vaccination. One intervention and control cluster
19	Mehta (2013) [93]	A randomized-controlled study evaluating a health-belief-model-based intervention to increase vaccination rates in college men. The reported outcome was intention to vaccinate.
10	O’Leary (2019) [94]	A cluster-randomized controlled study assessing the effectiveness of a multimodal intervention in obstetrics/gynecology clinics in increasing vaccination uptake. One intervention and control cluster.
21	Patel (2014) [95] NCT01343485 [96]	A cluster-randomized control study evaluating the impact of an automated reminders in increasing on-time completion of the three-dose HPV vaccine series. One intervention and control cluster.
22	Perez (2016) [2]	A randomized controlled study evaluating the effect of an information–motivation–behavioral skills (IMB) intervention in increasing HPV vaccination knowledge, motivation, and intentions among college-aged women. Reported outcome was intentions to vaccinate
23	Perkins (2015) [97]	A before–after study assessing the effectiveness of a provider-focused intervention in improving HPV vaccination rates in boys and girls. One intervention group and control group.
24	Rahman (2013) [98]	A cross-sectional study evaluating the impact of attending a well-woman clinic on HPV vaccine intent and uptake among both their sons and daughters. A cross-sectional study.
25	Rickert (2014) [99]	A before–after study assessing the impact of health beliefs on intent and first dose uptake of HPV vaccine among young adolescent males. One intervention and control cluster.
26	Roblin (2014) [100]	An observational study evaluating the influence of deductible health plans on receipt of the human papillomavirus vaccine series. An observational study.
27	Ruffin (2015) [101]	A retrospective study assessing the impact of electronic health record reminder on HPV vaccine initiation and timely completion among female patients. A retrospective study.
28	Russel (2012) [102]	A randomized controlled study assessing the effectiveness of text message reminders in improving vaccination appointment attendance and series completion among adolescents and adults. Abstract only.
29	Sanderson (2017) [103] NCT02808832 [104]	A cluster-randomized controlled study evaluating the effectiveness of provider-focused and patient-focused intervention strategies in increasing HPV vaccination. One intervention and control cluster.
30	Spleen (2012) [105]	A before–after study evaluating the impact of theory and community-based educational intervention on increasing parents’ HPV-related knowledge and parental intent to vaccinate their daughters against HPV. One intervention and control group.
31	Valdez (2015) [106]	A randomized controlled trial evaluating the effects of HPV vaccine education intervention on promoting informed decision-making about HPV vaccination among parents. Reported outcome were intentions to vaccinate
32	Whadera (2015) [107]	A prospective study assessing the effect of HPV educational intervention on HPV knowledge, vaccine acceptance, and vaccine series completion among female entertainment and sex workers. A prospective study.
33	Wedel (2016) [108]	A before–after study evaluating the effect of HPV educational intervention on increasing HPV vaccinations among military women. Not a controlled before and after study.
34	Wegwart (2014) [109]	A before–after study evaluating the effect of evidence-based HPV vaccination leaflets on understanding, intention, and actual vaccination decision. One intervention and control group
35	Whelan (2014) [110]	A retrospective study examining the relationship between school-based strategies and uptake of HPV vaccine. A retrospective study.
36	Winer (2016) [111]	A cluster-randomized controlled study evaluating the impact of an educational intervention on increasing HPV vaccination coverage in American Indian girls. One intervention and control cluster.
37	Zimmerman (2017) [112]	A before–after study evaluating the effect of the 4 Pillars™ Practice Transformation Program on improving adolescent HPV vaccination. One intervention and control group.
38	Zimmerman (2017) [113]	A cluster-randomized controlled study evaluating the effect of the 4 Pillars™ Practice Transformation Program on improving adolescent HPV vaccination. One intervention and control cluster.

**Table A4:** Risk of bias summary.

	Random Sequence Generation (Selection Bias)	Allocation Concealment (Selection Bias)	Blinding of Participants and Personnel (Performance Bias)	Blinding of Outcome Assessment (Detection Bias)	Incomplete Outcome Data (Attrition Bias)	Selective Reporting (Reporting Bias)	Other Bias
Bennett (2015) [22]							
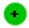
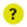
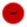
Brewer (2017) [23]							
Chao (2015) [24]							
Coley (2018) [25]							
Dempsey (2019b) [26]							
DiClemente (2015) [27]							
Fisher-Borne (2018) [30]							
Gilkey (2014) [28]							
Gilkey (2019) [29]							
Henrikson (2018) [31]							
Hofstetter (2017) [32]							
Hopfer (2012) [33]							
Irving (2018) [54]							
Joseph (2016) [34]							
Lee (2018) [35]							
Mantzari (2015) [36]							
Mclean (2017) [55]							
Parra-Medina (2015) [56]							
Patel (2012) [37]							
Pot (2017) [38]							
Rand (2015) [39]							
Rand (2017) [40]							
Reiter (2018) [41]							
Richman (2019) [42]							
Richman (2016) [43]							
Rickert (2015) [44]							
Suh (2012) [45]							
Szilagyi (2015) [46]							
Szilagyi (2013) [47]							
Tiro (2015) [48]							
Tull (2019) [49]							
Underwood (2019) [50]							
Vanderpool (2013) [51]							
Wilkinson (2019) [52]							
Zimet (2018) [53]							
